# Viral RNA in City Wastewater as a Key Indicator of COVID-19 Recrudescence and Containment Measures Effectiveness

**DOI:** 10.3389/fmicb.2021.664477

**Published:** 2021-05-17

**Authors:** Nathalie Wurtz, Alexandre Lacoste, Priscilla Jardot, Alain Delache, Xavier Fontaine, Maxime Verlande, Alexandre Annessi, Audrey Giraud-Gatineau, Hervé Chaudet, Pierre-Edouard Fournier, Patrick Augier, Bernard La Scola

**Affiliations:** ^1^Aix Marseille Univ, IRD, AP-HM, MEPHI, Marseille, France; ^2^Institut Hospitalo-Universitaire Méditerranée-Infection, Marseille, France; ^3^Bataillon de Marins-Pompiers de Marseille, Marseille, France; ^4^CAMGAU Consulting, Nice, France; ^5^Aix Marseille Univ, IRD, AP-HM, VITROME, Marseille, France

**Keywords:** COVID-19, SARS-CoV-2, sewers, wastewater, RNA

## Abstract

In recent years, and more specifically at the beginning of the COVID-19 crisis, wastewater surveillance has been proposed as a tool to monitor the epidemiology of human viral infections. In the present work, from July to December 2020, the number of copies of SARS-CoV-2 RNA in Marseille’s wastewater was correlated with the number of new positive cases diagnosed in our Institute of Infectious Disease, which tested about 20% of the city’s population. Number of positive cases and number of copies of SARS-CoV-2 RNA in wastewater were significantly correlated (*p* = 0.013). During the great epidemic peak, from October to December 2020, the curves of virus in the sewers and the curves of positive diagnoses were perfectly superposed. During the summer period, the superposition of curves was less evident as subject to many confounding factors that were discussed. We also tried to correlate the effect of viral circulation in wastewater with containment measures, probably the most unbiased correlation on their potential inflection effect of epidemic curves. Not only is this correlation not obvious, but it also clearly appears that the drop in cases as well as the drop in the viral load in the sewers occur before the containment measures. In fact, this suggests that there are factors that initiate the end of the epidemic peak independently of the containment measure. These factors will therefore need to be explored more deeply in the future.

## Introduction

In December 2019, an outbreak of coronavirus disease, further refered to as Covid-19, was detected in Wuhan, China (Al-Tawfiq, 2020; Huang et al., 2020; Rothan and Byrareddy, 2020; Toit, 2020). This epidemic is due to Severe Acute Respiratory Syndrome—Coronavirus 2 (SARS-CoV-2), which was classified as a new strain of coronavirus. WHO declared on March 11, 2020 a worldwide pandemic (World Health Organization, 2020b). To date, more than 136 million cases and more than 2.9 million deaths have been reported worldwide as of March 9, 2021 (World Health Organization, 2020a).

This new coronavirus resembles classical respiratory infection with common symptoms, including dry cough, fever, tiredness, myalgia and difficulty in breathing (Petersen et al., 2020).

SARS-CoV-2 is able to cause gastrointestinal symptoms in addition to respiratory symptoms, in approximately 2–10% of positive cases (Gao et al., 2020; Memish et al., 2020). Several recent studies reported the presence of SARS-CoV-2 RNA in stool and anal/rectal swabs feces, not only in symptomatic, but also in asymptomatic patients (Gu et al., 2020; Holshue et al., 2020; Song et al., 2020; Tang et al., 2020; Xiao et al., 2020). It has even been shown that virus in stools was still infectious (Dergham et al., 2020).

Setting up monitoring of virus levels in wastewater seemed thus logical. Indeed, wastewater-based epidemiology approach has already been used to follow disease outbreak, as previously demonstrated for enteric viruses, such as poliovirus or hepatitis virus (Asghar et al., 2014; Hellmér et al., 2014) and could also been used to monitor SARS-CoV-2 clusters (Carducci et al., 2020; Randazzo et al., 2020). Detection of SARS-CoV-2 RNA in wastewater samples has already been reported in many countries (Ahmed et al., 2020; Albastaki et al., 2020; Alpaslan Kocamemi et al., 2020; Bar Or et al., 2020; Haramoto et al., 2020; Hasan et al., 2020; La Rosa et al., 2020; Medema et al., 2020; Randazzo et al., 2020; Wang et al., 2020; Wu et al., 2020; Wurtzer et al., 2020).

In the present work, we evaluated the number of copies of SARS-CoV-2 RNA in Marseille wastewater and correlated these data with the number of new positive cases observed in Marseille at our Institute of Infectious Diseases since July 1, 2020. Correlation between these two indicators was made on the basis of daily observations and confronted with the effectiveness of the containment measures decreed by the national Ministry of Health throughout the crisis.

## Materials and Methods

### Sampling Sites and Wastewater Collection

**FIGURE 1:**
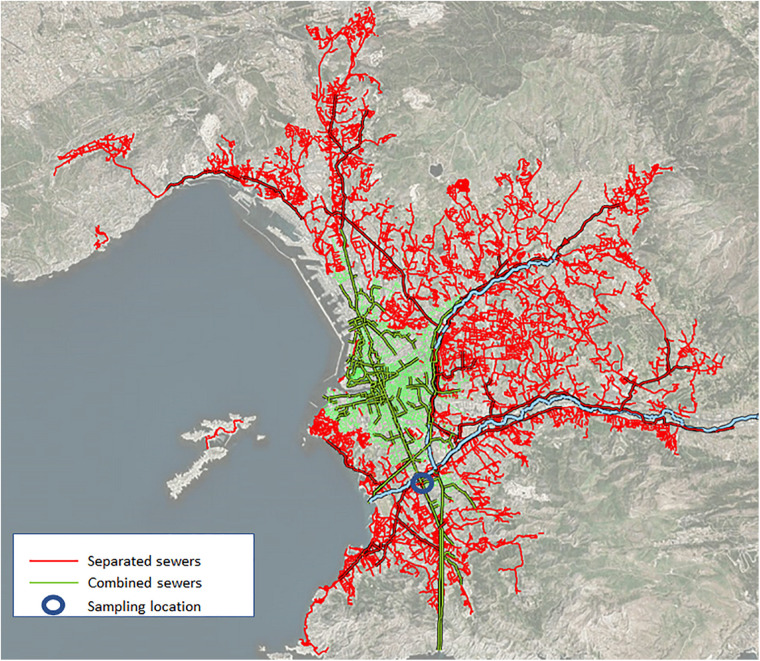
Wastewater networks in Marseille. The separate network (red lines) drains the major surface part of Marseille wastewater. The combined network (green lines), that contains a mixture of rainwater and wastewater, drains the city center. The blue circle represents the sampling point.

Wastewater was collected from two distinct sewer networks. The separate network (noted RS) drains the major surface part of Marseille wastewater and nearly all hospitals of the city, especially COVID-19 dedicated units (zone in red line in Figure 1). The number of inhabitants connected to this network is 614,623. The combined network (noted RU), that contains a mixture of rainwater and wastewater, drains the city center of Marseille (zone in green on the map and dark blue line in Figure 1), a place that concentrates most of the touristic activity of Marseille, including most restaurants and night festive life. The number of inhabitants connected to this network is 359,123.

The two samples of 250 ml each were collected by the SERAMM (Marseille Metropole Sanitation Departement) by 2 independent vacuum samplers “ASP-Station 2000 RPS20B” (Endress Hauser, Huningue, France). This type of sampler allows the filling of a refrigerated flask of 20 L per 24 h of wastewater collected from 8 a.m. to 8 a.m. The dates of collection were from July 1st, 2020 to December 15th 2020. Samples were transferred every day on ice to NRBC’s laboratory (NRBC unit—nuclear, radiological, biological, chemical) of the BMPM unit (Marseille Fire Brigade Battalion) and treated within 1 h of collection. Before treatment, they were stored at 4°C. In total over the period tested (i.e., 168 days), 117 wastewater samples were collected, which corresponds to approximately one sample every 1.4 days. SARS-CoV-2 copy number of RU and RS were totalized with adjustment to the respective population of their area when combined. An 8 days moving average was performed and the results were correlated with the SARS-CoV-2 Marseille positive cases.

### SARS-CoV-2 Virus Semi-Quantitative Evaluation in Wastewater

For detection of SARS-CoV-2 in wastewater, the BioFire^®^ COVID-19 Test (BioFire Defense, Salt Lake City, United States), a nested multiplexed real-time RT-PCR that detect 3 targets of SARS-CoV-2 genome, was used for qualitative detection of the virus according to the manufacturer’s intructions, using FilmArray Torch instrument (Biomérieux, Grenoble, France. The limit of detection (LoD) of the BioFire^®^ COVID-19 Test provided by the manufacturer (BioFire Defense, LLC) is 330 genomic copies per milliliter. To control this LoD, serial dilutions of known copies of synthetic SARS-CoV-2 RNA (SARS-CoV-2 Standard COV019, Biorad France, 200,000 copies/ml) were performed, from 2,000 genomic copies/ml to 50 copies/ml. Five technical replicates were performed at each dilution. Interpretation was made according to manufacturer’s instructions and based on melt curve analysis as follows: “positive” when at least 2 out of 3 targets were detected, “negative” when no target was detected and “equivocal” when 1 target was detected. For the semi-quantitative evaluation of SARS-CoV-2 virus in wastewater, twofold serial dilutions of the wastewater were performed. The last positive reported dilution as defined above indicates the number of copies per ml. The wastewater data were compared against community infection data evaluated in our institute^1^.

### Factors of Variation Analyzed

The daily mean temperature and amount of rain (Supplementary Table 1) that can affect the number of positive SARS-CoV-2 cases or copy numbers were analyzed. The different measures implemented by the French government analyzed herein were obligation to wear a mask in confined area, obligation to wear a mask everywhere in Marseille, total closure of bars and restaurants in Marseille, re-opening of bars and restaurants in Marseille, reduction to 50% of the presence of student in universities, implementation of the curfew and implementation of the lockdown (Supplementary Table 1).

### Statistical Analysis

The concordance between the time series has been studied using cross-correlation for finding the lag between the curves and co-integration using the Johansen procedure (Johansen, 1991), after having tested the presence of unit roots with the Elliott, Rothenberg and Stock unit root test (Elliott et al., 1996). Using co-integration allows avoiding the risk of detecting a spurious correlation between the times series due to their on-stationarity. Statistical processes were done on R (v 4.0.2).

## Results

### Validation of the Limit of Detection (LoD)

Verification of LoD showed that at 2,000 and 600 copies, all replicates were positive with 2 or 3 genes detected (Table 1). At 400 copies, 1 replicate was positive for all 3 targets, whereas 3 replicates were positive for 2 targets and one was equivocal. For 300 copies, 3 replicates were positive for two targets and 2 replicates were equivocal. Below 300 copies, all sample tested were equivocal or negative. Thus, the LoD where all samples are detected is 300 genomic copies/ml in perfect agreement with manufacturer’s data. This value was used as our reference for further analyses.

**TABLE 1:** Results of the BioFire COVID-19 using serial dilutions of synthetic SARS-CoV-2 RNA.

**Biofire LoD**	**Genomic copies/ml**	**Number of replicates**	**Number of target detected**			
			**3/3 positive**	**2/3 positive**	**1/3 equivocal**	**0/3 negative**
0.15	50	5	0	0	0	5
0.3	100	5	0	0	2	3
0.6	200	5	0	0	4	1
0.9	300	5	0	3	2	0
1.2	400	5	1	3	1	0
1.8	600	5	3	2	0	0
6	2,000	5	5	0	0	0

### Evolution of SARS-CoV-2 Quantification in Wastewater and COVID-19 Cases During the 6 Months of Survey

From July 1st to September 1st, the amount of virus in the sewer increases to reach a mean of almost 6,000 copies/ml. Then, the amount of virus in the sewer dropped to an average of approximately 1,000 copies/ml from September 1 to September 23. From September 24, the level of virus increased rapidly with a peak on October 22, with a quantity of 9,000 copies/ml on that day. Subsequently, a decrease in the amount of virus was observed in wastewater reaching < 300 copy/ml.

**FIGURE 2:**
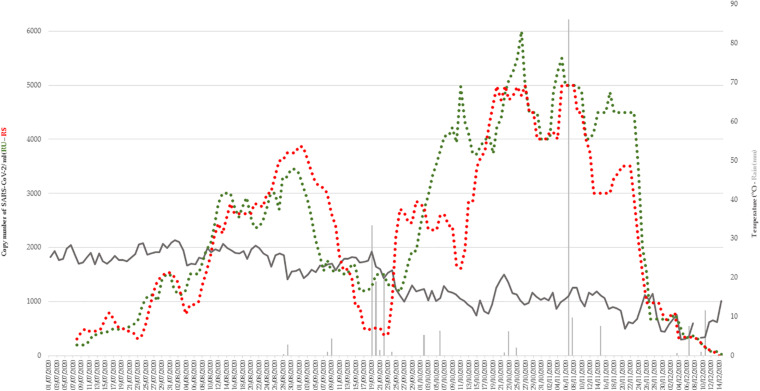
Variations in mean outdoor temperature (dark gray) and rain fall (light gray) during the period of study (from July 1 to December 15). Copy number of SARS-CoV-2 in RU (green) and RS (red) wastewater networks were represented.

Variations in mean outdoor temperature (ranging between 4.1 and 29.5) had no effect on the number of SARS-CoV-2 copy numbers in wastewater (Figure 2). There was 2 episodes of rain from September 19 to 22 and November 7 to 8. The quantity of viruses did not drop during the first episode, but possibly with the second.

**FIGURE 3:**
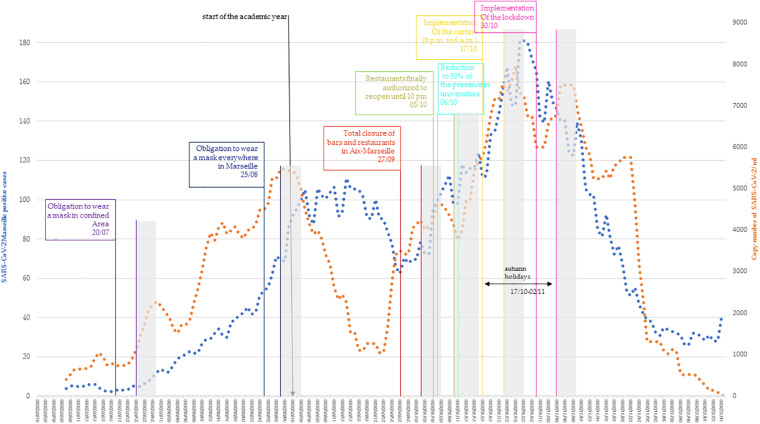
Correlation of SARS-CoV-2 Marseille new positives cases (blue curve) and copy number of SARS-CoV-2 in RU + RS wastewater networks (orange curve). Eight-days moving average was represented. The measures implemented by government were positioned at the day of application. In gray, the period where efficiency of the measures can be observed.

The daily number of new cases of SARS-CoV-2 in Marseille detected at the IHU using an 8 days moving average was analyzed and is represented on Figure 3. Since July 1st, the number of positive cases has been slowly increasing until reaching a plateau in September 2020 with an average number of positive cases of about 100 per day. This plateau is grossly observable during the first 3 weeks of September. Then, for 1 week, the number of positive cases decreased with a minimum average number of positive cases of about 60. From September 28, a rapid increase in the number of positive cases was observed, peaking on October 26 with a maximum of 303 positive cases on that day. From this date, the number of positive cases decreases considerably, reaching an average of 20 positive cases in the first weeks of December.

When looking at the evolution between the number of SARS-CoV-2 copies in wastewater and the number of SARS-CoV-2 positive cases, from July 1st to September 1st, the amount of virus in the sewer increases as does the number of positive cases. Then, while the number of positive patients stagnates, the amount of virus in the sewer drops from September 1st to September 23rd. In this phase, a discrepancy between the number of SARS-CoV-2 positive cases and the amount of virus in the wastewater was observed. From September 24, a perfect correspondence was observed between the number of positive cases and the amount of virus observed in the sewers, with a peak observed on October 22. Then, a decrease in the amount of virus was observed in wastewater, in agreement with the decrease in the number of SARS-CoV-2 positive cases. The cross-correlation testing of the curves shows a maximal correlation with a lag = 0 (*r* = 0.648, *p* < 0.01). Testing these curves with the Johansen procedure rejects the null hypothesis of no cointegration with *p* = 0.013, proving that there is a statistically significant connection between the two curves.

### Correlation Between SARS-CoV-2 Quantification in Wastewater and Containment Measures Effectiveness

The different measures implemented by the French government with precise dates were correlated with the evolution of the cases and the concentration of the virus in wastewater (Figure 3), and their effect was tentatively assessed between 5 and 10 days after their implementation (in gray). The first measure implemented during summer was the mandatory wearing of a mask in confined areas on July 20. Five to ten days after this was introduced, the number of SARS-CoV-2 positive cases continues to increase slowly, as does the number of SARS-CoV-2 copies in wastewater. The 25th of august, to wear a mask became mandatory everywhere in Marseille. Five to ten days later, the number of SARS-CoV-2 positive cases continued to increase, while the number of SARS-CoV-2 copies stagnated and began to decline. The 27th of September, bars and restaurants in Marseille were ordered to close. Five to ten days later, both level of positive cases and copy number of SARS CoV-2 first slowly decreased, and then rapidly increased. Finally, the bars and restaurants reopened on October 5 and on October 6, and universities reduced their capacity by 50%. The curve for positive cases and wastewater SARS-CoV-2 copy numbers continued to increase rapidly. The 17th of October, a curfew was implemented from 9 p.m. to 6 a.m. This curfew coincided to the fall vacations for the children and therefore the closure of schools for 2 weeks. Five to ten days later, the number of positive cases reached a peak and the copy number of SARS-CoV-2 started to decrease. Just after this period, a lockdown was implemented (October 30). First, immediately after the implementation of the lockdown, the decline in Covid cases and in the copy number of SARS-CoV-2 stopped abruptly and, paradoxically, increased before the downtrend resume. Following this period, the drop in the number of positive cases and the amount of virus in sewers continued, reaching a level roughly equivalent to that of early August for cases and early July for wastewater.

## Discussion

Recently, several studies have explored the detection and quantification of SARS-CoV-2 in wastewater around the world, such as in the Netherlands (Medema et al., 2020), France (Wurtzer et al., 2020), United States (Wu et al., 2020), Australia (Ahmed et al., 2020), Italy (La Rosa et al., 2020), and Germany (Westhaus et al., 2021). However, few have attempted to establish a correlation between viral load in the wastewater and the number of infected patients (Ahmed et al., 2020; Chavarria-Miró et al., 2021; Medema et al., 2020; Randazzo et al., 2020; Trottier et al., 2020; Vallejo et al., 2020; Wu et al., 2020; Wurtzer et al., 2020).

In the present work, a correlation between the quantitative detection of SARS-CoV-2 in wastewater and the number of cases diagnosed in our institute was observed. The BioFire FilmArray System is PCR assays that combines all the reagents necessary for sample extraction (including extraction controls), reverse transcription, PCR (and associated positive and negative controls). Indeed, the Biofire system possess two control systems, a RNA and a DNA controls that verify the quality of the extraction, reverse transcription and PCR. The RNA process control assay targets an RNA transcript from the yeast *Schizosaccharomyces pombe*. The yeast is present in the pouch in a freeze-dried form and becomes rehydrated when sample is loaded. The control material is carried through all stages of the test process, including lysis, nucleic acid purification, reverse transcription, PCR1, dilution, PCR2, and DNA melting. A positive control result indicates that all steps carried out in the BioFire COVID-19 Test were successful. The PCR2 control assay detects a DNA target that is dried into wells of the array along with the corresponding primers. A positive result indicates that PCR2 was successful. Both control assays must be positive for the test run to pass. Using endpoint melting curve data, the BioFire System software automatically analyzes the results for each target. In practice, it is a very sensitive and very specific detection system with its own controls which has only two major flaws, its excessively high cost and the impossibility of carrying out a quantitative evaluation. It is for this reason of using successive dilutions in order to make a semi-quantitative evaluation of the number of copies and to verify its LoD using a calibrated control. The Biofire system, which is not usually used in this application, appeared to be effective and the LoD supplied by the manufacturer was in agreement both with our titration controls and with the previous studies (Liotti et al., 2020). This does not mean that the absolute copy number was absolutely correct, because there are possible inhibitors or interfering substances in the wastewater (Shieh et al., 1995; Haramoto et al., 2020), but it still allowed us to study the kinetics of viral circulation. As for prolonged excretion of SARS-CoV-2 in feces, it is a variable that is impossible to control and which is common to all works of the same type. In addition, prolonged carriage (beyond a week) is above all the prerogative of immunocompromised patients, therefore in the minority in the population. Finally, the use of an 8 days moving average makes it possible to smooth out this type of variation. With the virus circulating at a high level, virus concentration was always well above the biofire LoD. But it is likely that at the end of the current epidemic episode, when the virus level in wastewater will have dropped, a concentration step will be necessary. As for COVID-19 cases, the number was based on people attending our institute, which represents 20% of all SARS-CoV-2 tests carried out for the whole city during the period of the present study^2^. While other private sites, even during the summer, had deadlines for making appointments and reporting results ranging from 2 to 5 days, by modifying our organization (Bouam et al., 2021; Fournier et al., 2021), all people arriving at the institute without an appointment between 7 a.m. and 7 p.m. obtained their results in less than 12 h, on their mobile phone if they have one, or by picking it up at the institute. Thus, the rates observed in our institute were representative of the evolution of the epidemic in real time.

Throughout the study period, i.e., from July to mid-December, three types of curves and correlations between the levels of SARS-CoV-2 in wastewater and the number of cases could be observed. The statistical analysis of these two curves shows that during the study period they exhibit a concordant evolution with no temporal lag. A longer study period would be preferable but of more than fifty articles published on the survey of SARS-CoV-2 in wastewater samples, only 4 covers such a long period (Trottier et al., 2020; Agrawal et al., 2021; Prado et al., 2021; Saguti et al., 2021). During the third part of the period, roughly from the end of September to the end of November, there was a perfect correlation in the kinetics of the two curves with a variable shift on the accelerations or decelerations remaining very moderate (Figure 3). This trend of correlation between SARS-CoV-2 rate in wastewater and number of positive patients has been observed in other studies (Medema et al., 2020; D’Aoust et al., 2021). It was only at the beginning of December that there was a real dissociation with the disappearance of SARS-CoV-2 below the LoD while the number of cases remained relatively high. This was associated with massive testing before Christmas (see Supplementary Figure 1; Roser et al., 2020). The aim was to detect as many cases as possible in order to avoid an explosion of cases after the holiday season for fear of a rebound like the one observed after Thanksgiving in California (Fernandez et al., 2020; Mehta et al., 2020).

During the first period, the picture was clearly different. From the beginning of July to the beginning of September, i.e., exactly at the peak of the tourist season, the correlation was also observed, but in a very different way. The copy number of SARS-CoV-2 and the number of cases increased in a linear and perfectly parallel manner. In contrast, unlike in the third period, the rate of viruses in wastewater was comparatively higher than in the last period. This discrepancy could be due to the fact that the increase in wastewater precedes the appearance of signs in patients for a longer period of time during the summer period. Indeed, in other studies, it has been observed that the increase in wastewater rates precedes the increase in cases by 4 days to several weeks (Ahmed et al., 2020; La Rosa et al., 2020; Medema et al., 2020; Randazzo et al., 2020; Trottier et al., 2020). The particularity during the summer was also that the tourists invested in masse the city during the day and part of the night for visits, then the restaurants and nightclubs (some received up to 3,000 people simultaneously), but they did not stay there permanently. They could therefore emit the virus in the toilets in quantity but were mostly not tested in Marseille. This type of discrepancy would certainly be avoidable by continuously measuring the effluent flow rate and not considering that this flow rate is always the same, as if true for a constant population, it can change during periods of high tourist activity. Another hypothesis could be that patients were less symptomatic in summer and therefore less tested. This has been verified in other works and other viral diseases (Shaman et al., 2018; Jones et al., 2020) and may be partly related to the fact that those infected during the summer were on average much younger. Indeed, younger subjects are both less symptomatic and less inclined to be tested with minor symptoms (Kronbichler et al., 2020; Gautret et al., 2021). The last period of interest is intermediate, spanning approximately the month of September. It corresponds to the time of the departure of the tourists from the city, the return of the inhabitants and the beginning of the school year. During this period, there is a real dissociation between the rates of virus in the sewers, which drop sharply before gradually increasing, while the number of positive cases remains stable. This shift was due to interfering factors, such as temperature or precipitation, but found no correlation (Figure 2). There is a time lag between case and virus in the sewers, which is comparable to that observed in the first phase but in reverse. In conclusion, there are two really different episodes, the epidemic from early July to early September, where the difference between the wastewater virus and the case of COVID-19 is significant, then the period from the beginning of October to the end of December, where a real correlation exists. Besides the period (great summer heat) and the different habits (nightlife and more visitors), the last thing that was different over these periods is the distribution of the majority strains. While during the summer the Marseille 1 genotype was predominant, it was the Marseille 4 genotype that predominated over the second period (Colson et al., 2020, 2021). The lower severity of the infections linked to this Marseille 1 variant is the last possible hypothesis that could explain this difference (Colson et al., 2020).

In a second part of this work, the levels of SARS-CoV-2 RNA in wastewater, the number of newly diagnosed COVID-19 patients and the different measures implemented by the French government were tentatively correlated. The effect of the formers was evaluated between 5 and 10 days after their application. This delay corresponds to the COVID-19 mean incubation period, with a median of 4–5 days from exposure to symptoms, but can be extended to 14 days (Lauer et al., 2020). This incubation period is also similar with that of other known human coronaviruses, including SARS (Varia et al., 2003), MERS (Virlogeux et al., 2016), and non-SARS human coronavirus (Lessler et al., 2009). The first measure implemented was the obligation to wear a surgical mask, first in confined areas and then, 1 month later, everywhere in Marseille. Herein, the number of new COVID-19 infected persons continued to increase despite wearing a mask. This measure seems to be not effective against the spread of COVID-19, but it is also obvious that the wearing of a mask when the outside temperature exceeds 30°C is not bearable and is therefore not worn properly. The second measure implemented was the closure of bars and restaurants at the end of September, mostly on the basis of two American studies that pointed the role of restaurants in contamination (Fisher et al., 2020; Chang et al., 2021). In France, the ComCor study was conducted last October on the places and circumstances of new contaminations and concluded that the highest risk of transmission of the virus occurs during meals, whether they take place in the private sphere (family, friends) or in public places (cafes, restaurants…) (Galmiche et al., 2020). In our study, the number of COVID-19 positive cases continued to increase despite the total closure of bars and restaurants. Another French study was in agreement with our results, stating that the measures implemented since September 23rd (in particular the closure of bars and restaurants) had no effect in the weeks that followed (Spaccaferri et al., 2020). The third measure implemented was the curfew from 9 p.m. to 6 a.m. There is little data on the effectiveness of the curfew. A French study compared the evolution of SARS-CoV-2 epidemic (second episode) in departments where the curfew was implemented and not (Spaccaferri et al., 2020). The study reported that the departments that have not been subject to curfew are those that have been only slightly or not affected by this epidemic phase. There was indeed a slight decrease in the number of positive cases, but just after the number of positive cases had peaked. The last measure implemented by the French government was the lockdown on October 30. Our study shows that, in our city, lockdown does not play a role in slowing down the rate of contamination. Nor is it associated with death rates in all countries affected by the pandemic, and no one has, to date, provided scientific proof of its long-term benefits (De Larochelambert et al., 2020). The number of positive cases began to clearly decrease before the lockdown was implemented. Therefore, although this measurement may have had an effect in our country on the rate of reduction or elsewhere on peak height, it was not the cause. Furthermore, given the very low level of seroprevalence, for which it is inconceivable that this reduction is the effect of herd immunity (Sisay and Tolessa, 2020), there are clearly other factors to be investigated. In 2002, the SARS-CoV-2 epidemic began and lasted about a year and a half, infecting at least 8,000 people and killing 10% of them (Davis, 2020). Although it mainly affected East Asian countries, by its end, SARS had spread throughout the world. It is accepted that the epidemic was contained by strict quarantine measures in front of a symptomatic viral disease that allowed for the rapid identification and isolation of cases. In this widespread self-satisfaction over our ability to end a pandemic, no one has tried to understand whether this disappearance could be linked to factors other than strict quarantine measures. The results that we report here on the fact that the epidemic linked to the majority clone of the period begins to decrease independently of these measures suggest that for SARS-CoV-2, other factors than containment measures, may play a role. Identifying them could have a major effect on the control of the current pandemic and should avoid the most restrictive that were not proven to be efficient as compared to less restrictive (Bendavid et al., 2021).

## Data Availability Statement

The raw data supporting the conclusions of this article will be made available by the authors, without undue reservation.

## Ethics Statement

The studies involving human participants were reviewed and approved by the Nasopharyngeal samples were done at the IHU Mediterranean infection as part of COVID-19 diagnosis and follow-up of patients. The study was approved by the ethical committee of the University Hospital Institute Méditerranée Infection (No: 2020-029). Informed consent was obtained from all subjects involved in the study. Written informed consent for participation was not required for this study in accordance with the national legislation and the institutional requirements.

## Author Contributions

AL, PA, BL, and AD: conceptualization. NW and AL: methodology and writing-original draft preparation. BL and PA: validation, project administration, and funding acquisition. NW, AL, and AG-G: formal analysis. NW, AL, PJ, XF, MV, and AA: investigation. BL and P-EF: writing-review and editing. All authors have read and agreed to the published version of the manuscript.

## Conflict of Interest

AD was employed by the company CAMGAU Consulting. The remaining authors declare that the research was conducted in the absence of any commercial or financial relationships that could be construed as a potential conflict of interest.

## Footnotes

1 https://www.mediterranee-infection.com/

2 geodes.santepubliquefrance.fr
