# Supplementary material for: Viral RNA in City Wastewater as a Key Indicator of COVID-19 Recrudescence and Containment Measures Effectiveness
Source: Front Microbiol. 2021 May 17;12:664477. doi: 10.3389/fmicb.2021.664477 (PMC8165276; doi:10.3389/fmicb.2021.664477)
Supplement: Supplementary file 1 [file Data_Sheet_1.PDF]

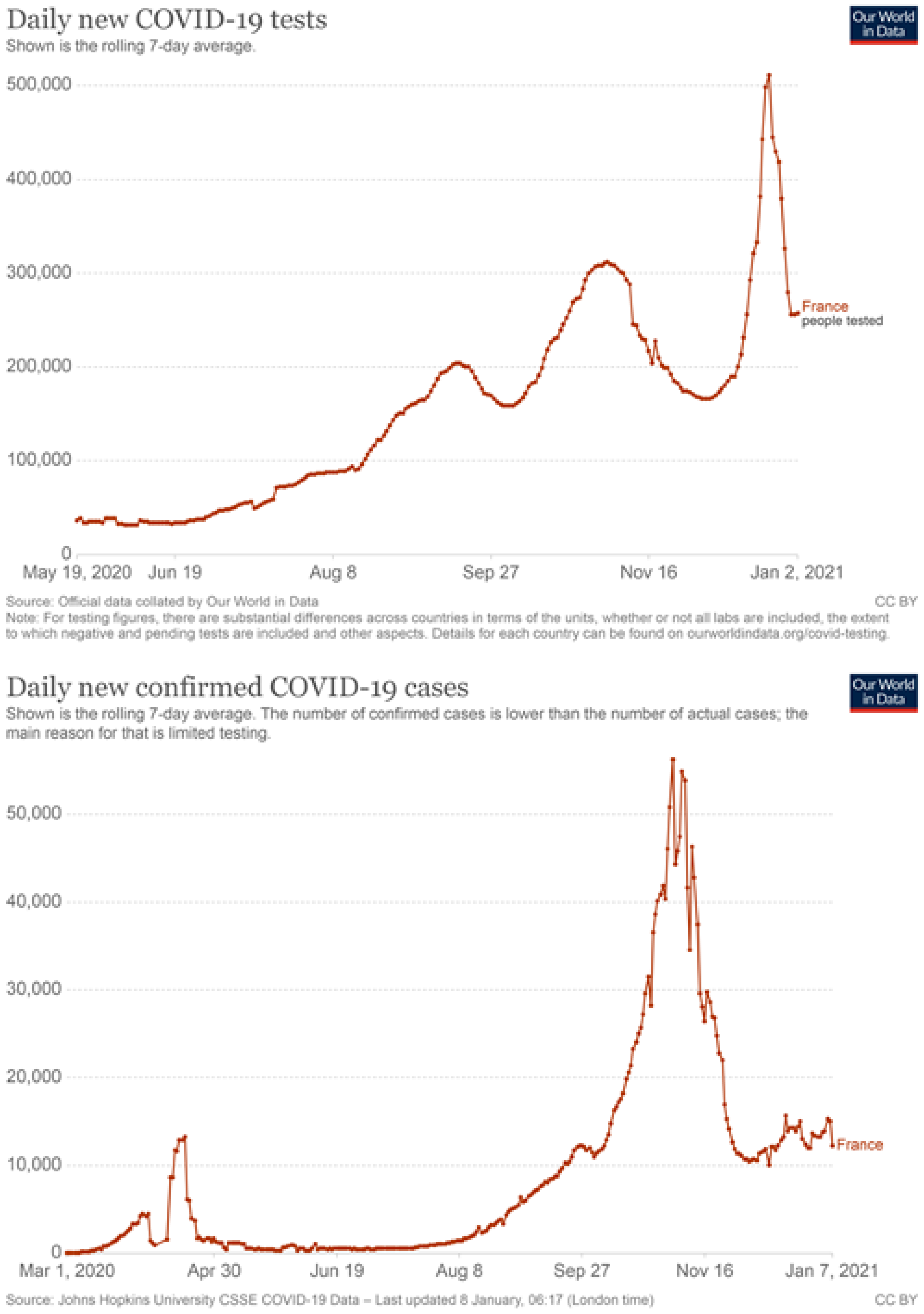

Figure S1. Correlation of daily new COVID-19 tests and daily new confirmed COVID-19 cases in France. These data are available online https://ourworldindata.org/coronavirus
