# Supplementary material for: Viral RNA in City Wastewater as a Key Indicator of COVID-19 Recrudescence and Containment Measures Effectiveness
Source: Front Microbiol. 2021 May 17;12:664477. doi: 10.3389/fmicb.2021.664477 (PMC8165276; doi:10.3389/fmicb.2021.664477)
Supplement: Supplementary file 2 [file Table_1.DOCX]

**Table S1**. Factors of variation that can affect the number of positive SARS-CoV-2 cases or copy numbers in wastewater.

| **Factors / measures** | **Web link** |
| --- | --- |
| Daily mean temperature and amount of rain | https://www.meteo60.fr/stations-releves/station-mois?station_id=07650&mois=11&annee=20 |
| Obligation to wear a mask in confined area | https://www.legifrance.gouv.fr/download/file/6jSpA0cWPxkOQ3AuvS7vvBqAan03mhLJC5z3cVMEAsc=/JOE_TEXTE |
| Obligation to wear a mask everywhere in Marseille | https://www.bouches-du-rhone.gouv.fr/content/download/37966/215784/file/Covid-19%C2%A0%20renforcement%20des%20mesures%20pour%20lutter%20contre%20la%20propagation%20du%20virus%20dans%20les%20Bouches-du-Rh%C3%B4ne.pdf |
| Total closure of bars and restaurants in Marseille | http://www.bouches-du-rhone.gouv.fr/content/download/38428/218472/file/Arr%C3%AAt%C3%A9%20n%C2%B0180%20du%2027%20septembre%202020%20OK-.pdf |
| Re-opening of bars and restaurants in Marseille | https://www.bouches-du-rhone.gouv.fr/content/download/38563/219477/file/C.P.%20Covid-19%C2%A0%20r%C3%A9ouverture%20des%20restaurants%20%C3%A0%20Aix-en-Provence%20et%20Marseille.pdf |
| Reduction to 50% of the presence of student in universities | https://services.dgesip.fr/fichiers/Circulaire_Renforcement_des_consignes_sanitaires_en_zones_d_alerte_renforcee_et_maximale.pdf |
| Implementation of the curfew | https://medias.amf.asso.fr/upload/files/D%C3%A9cret_17_octobre.pdf |
| Implementation of the lockdown | https://www.legifrance.gouv.fr/download/file/YTXQyL3I14RgMkscchJ4EWWUgvYvfJ3GciREwkWtl3E=/JOE_TEXTE |
